# Multicenter Study of Intense Pulsed Light for Patients with Refractory Aqueous-Deficient Dry Eye Accompanied by Mild Meibomian Gland Dysfunction

**DOI:** 10.3390/jcm9113467

**Published:** 2020-10-28

**Authors:** Reiko Arita, Shima Fukuoka, Takanori Mizoguchi, Naoyuki Morishige

**Affiliations:** 1 Department of Ophthalmology, Itoh Clinic, 626-11 Minami-Nakano, Minumaku, Saitama, Saitama 337-0042, Japan; 2 Lid and Meibomian Gland Working Group (LIME), Tokyo 112-0006, Japan; fshima3271@gmail.com (S.F.); t-mizo@siren.ocn.ne.jp (T.M.); morishig@corneajp.com (N.M.); 3 Omiya Hamada Eye Clinic, 1-169-1, Sakuragicho, Omiyaku, Saitama 330-0854, Japan; 4 Mizoguchi Eye Clinic, 6-13, Tawaramachi, Sasebo, Nagasaki 857-0016, Japan; 5 Division of Cornea and Ocular Surface, Ohshima Eye Hospital, 11-8, Kamigofukumachi, Hakataku, Fukuoka 812-0036, Japan

**Keywords:** aqueous-deficient dry eye, meibomian gland dysfunction, meibomian gland, intense pulsed light

## Abstract

Aqueous-deficient dry eye (ADDE) and meibomian gland dysfunction (MGD) can be refractory to therapy. Intense pulsed light (IPL) was recently introduced as an effective treatment for MGD. We here evaluated the efficacy of IPL combined with MG expression (MGX) compared with MGX alone (*n* = 23 and 20, respectively) for patients with refractory ADDE with mild MGD at three sites. Symptom score, visual acuity (VA), noninvasive breakup time (NIBUT) and lipid layer thickness (LLT) of the tear film, lid margin abnormalities, fluorescein BUT (FBUT), fluorescein staining, tear meniscus height (TMH), meibum grade, meiboscore, and Schirmer’s test value were assessed at baseline and 1 and 3 months after treatment. LLT, plugging, vascularity, FBUT and NIBUT were improved only in the IPL-MGX group at three months compared with baseline. All parameters with the exception of VA, meiboscore, TMH, Schirmer’s test value were also improved in the IPL-MGX group compared with the control group at three months, as was VA in patients with central corneal epitheliopathy. Although IPL-MGX does not affect aqueous layer, the induced improvement in quality and quantity of the lipid layer may increase tear film stability and ameliorate symptoms not only for evaporative dry eye but for ADDE.

## 1. Introduction

Dry eye disease is a common condition that causes ocular discomfort [1]. Although it generally does not reduce conventionally tested visual acuity (VA), most individuals with this condition manifest impairment of functional visual acuity, with higher-order aberrations in particular leading to disturbance of quality of vision [2]. Dry eye disease is classified into two major subtypes—aqueous-deficient dry eye (ADDE) and evaporative dry eye (EDE) [3]—both of which can involve pathology of meibomian glands, lacrimal glands, the eyelids, the tear film, and ocular surface cells [4]. ADDE and EDE tend to coexist, in part because the lacrimal gland defects associated with ADDE can lead to meibomian gland dysfunction (MGD) [5], and consequent EDE as a result of friction between the lid margin and the cornea and conjunctiva [6].

Common therapies for patients with ADDE include various topical medications such as cyclosporine, diclofenac sodium, steroids, loteprednol etabonate, resolvin E1, tacrolimus, autologous serum, and vitamin A [7,8]. In addition, punctal plug insertion, oral systemic antibiotics, surgery, dietary modification, local environmental changes, and alternative medicines have been applied [8]. Common therapies for patients with MGD, which is a major cause of EDE, include the application of a warm compress [9], the practice of lid hygiene [9], dietary supplementation with omega-3 fatty acids [9], forced meibum expression [9], intraductal probing [10], automated thermal pulsation [11], and the administration of topical steroids [9], topical and oral antibiotics including topical cyclosporine and azithromycin [9], preservative-free artificial tears [9], lipid-containing eyedrops [9], and topical diquafosol [12,13]. Despite the varied treatment options available, however, some patients with ADDE or MGD are refractory to therapy and therefore do not experience complete or long-term relief of symptoms.

Intense pulsed light (IPL) therapy based on the delivery of intense pulses of noncoherent light with wavelengths of 500 to 1200 nm has been applied in dermatology to treat various conditions, including benign cavernous hemangiomas or venous malformations, telangiectasia, port wine stains, and other pigmented lesions [14,15]. The efficacy of IPL therapy for patients with dry eye due to MGD was discovered during IPL treatment of facial rosacea [16]. Subsequent studies found that IPL, with or without concomitant meibomian gland expression (MGX), is effective for improvement of subjective symptoms and objective findings in patients with mild to moderate MGD or dry eye [17,18,19,20,21,22,23,24,25,26,27,28]. The combination of IPL and MGX was also shown to be effective in patients with refractory MGD [28,29]. In addition, it ameliorated dry eye symptoms and improved meibomian gland function in patients with refractory dry eye, including not only individuals with MGD but also those with graft-versus-host disease or Sjögren syndrome [20] or those with keratoconjunctivitis sicca [30]. However, as far as we are aware, no previous study has evaluated the effects of IPL on tear fluid–related parameters in addition to meibomian gland–related parameters in patients with refractory ADDE accompanied by mild MGD. We have therefore now performed a multicenter, retrospective, controlled study to evaluate the efficacy of IPL combined with MGX in comparison with MGX alone in patients with refractory ADDE and mild MGD who had been treated with conventional therapies.

## 2. Experimental Section

### 2.1. Patients

This retrospective controlled study was approved by the Institutional Review Boards of Itoh Clinic (approval code: IRIN201903-03), Mizoguchi Eye Clinic, and Ohshima Eye Hospital on March 11th, 2019, and it adhered to the tenets of the Declaration of Helsinki. Patients with refractory ADDE associated with mild MGD who were treated with either IPL and MGX or MGX alone between April and December 2017 at three sites in Japan (Itoh Clinic, Mizoguchi Eye Clinic, and Ohshima Eye Hospital) were enrolled in the study. Informed consent to study participation was obtained from each patient.

Inclusion criteria were as follows: (1) an age of at least 20 years; (2) a diagnosis of ADDE based on the diagnostic criteria for ADDE in Japan [31], which encompass ocular symptoms, a fluorescein tear film breakup time (FBUT) of ≤5 s, a Schirmer’s test value of ≤5 mm, and the presence of conjunctival or corneal epithelial damage as evidenced by a fluorescein staining (Fluo) score of ≥1 (on a scale of 0 to 9) according to the van Bijsterveld method [32]; (3) a diagnosis of mild MGD based on the diagnostic criteria for MGD in Japan [33], which encompass ocular symptoms, plugged gland orifices (plugging grade [34] of ≥1, which corresponds to plugging of fewer than three gland orifices with a distribution of less than half of the full length of the lid), vascularity and irregularity of lid margins, reduced meibum expression (meibum grade of ≥2, on a scale of 0 to 3, where 0 = clear meibum easily expressed, 1 = cloudy meibum expressed with mild pressure, 2 = cloudy meibum expressed with more than moderate pressure, and 3 = meibum cannot be expressed even with strong pressure) [35], and a meiboscore of ≥3 (on a scale of 0 to 6) [36]; (4) refractoriness of ADDE as defined by the failure to respond over a period of ≥2 years to at least three types of conventional therapy prescribed in Japan, including tear replacement therapy, tear conservation, and anti-inflammatory eyedrops; and (5) a Fitzpatrick skin type of 1 to 4 based on sun sensitivity and appearance [37]. Exclusion criteria included the presence of active skin lesions, skin cancer, or other specific skin pathology or of active ocular infection or ocular inflammatory disease.

### 2.2. Experimental Design

Each patient underwent a series of four IPL-MGX treatment sessions or four sessions of MGX alone at 3-week intervals and was subjected to clinical assessment as described below both before treatment as well as 4 and 12 weeks after the final treatment session. All patients were asked to continue their current ocular medications as well as not to initiate therapy with a new topical or systemic agent for dry eye or MGD during the treatment course.

### 2.3. Clinical Assessment

The thickness of the lipid layer of the tear film (LLT), noninvasive breakup time of the tear film (NIBUT), and interferometric fringe pattern of the tear film were determined with a DR-1α tear interferometer (Kowa, Tokyo, Japan) as described previously [38]. Lid margin abnormalities (plugging of meibomian gland orifices and vascularity of lid margins) [34], FBUT, the Fluo score [32], tear meniscus height (TMH) based on fluorescein staining, and meibum grade [35] were evaluated with a slitlamp microscope. For determination of TMH, the center of the lower tear meniscus stained with fluorescein was photographed with a CCD camera attached to the slitlamp microscope, with a magnification of 10 × and under lighting with a blue-free filter. The photographs were examined by an ocular surface expert (R.A.) for semiquantitative grading of TMH as low, normal or high. Morphological changes of meibomian glands were assessed on the basis of the meiboscore [36] as determined by noninvasive meibography. Tear fluid production was measured by Schirmer’s test as performed without anesthesia [39]. Symptoms were assessed with the standard patient evaluation of eye dryness (SPEED) validated questionnaire (scale of 0 to 28) [40,41]. VA was also measured with the use of Landolt C charts, and best corrected Landolt VA was converted to logarithm of the minimum angle of resolution (logMAR) VA.

### 2.4. IPL-MGX Procedure

Before the first treatment, each patient underwent Fitzpatrick skin typing [37] and the IPL machine (M22; Lumenis, Yokneam, Israel) was adjusted to the appropriate setting (Toyos setting: 590-nm cutoff filter, triple pulses of 6.0 ms with an interval of 50 ms, and total fluence range of 13 to 15 J/cm^2^). At each treatment session, both eyes of the patient were closed and sealed with IPL-Aid disposable eye shields (Honeywell Safety Products, Smithfield, RI, USA). After generous application of ultrasonic gel to the targeted skin area, each patient received ~13 pulses of light (with slightly overlapping applications) from the right preauricular area, across the cheeks and nose, to the left preauricular area, reaching up to the inferior boundary of the eye shields. This procedure was then repeated in a second pass. Immediately after the IPL treatment, MGX was performed on both upper and lower eyelids of each eye with an Arita Meibomian Gland Compressor (Katena, Denville, NJ, USA). Pain was minimized during MGX by the application of 0.4% oxybuprocaine hydrochloride to each eye. Patients in the control group underwent the MGX procedure alone, without IPL.

### 2.5. Statistical Analysis

Data were found to be non-normally distributed with the Shapiro–Wilk test (*p* < 0.05), and nonparametric testing was therefore applied. The Mann–Whitney U test was used to compare numerical variables between the control (MGX alone) and the IPL-MGX groups. The Wilcoxon signed-rank test was used to compare numerical variables between baseline and either 1 or 3 months after the final treatment session. Fisher’s exact test was used to compare categorical variables between the control and IPL-MGX groups. The chi-square test was used to compare lipid layer grade and TMH between before and either 1 or 3 months after the final treatment session. Adjusted *p* values were calculated by multiplication of obtained *p* values by the number of comparisons in Bonferroni’s correction. The outcome variables of the study were the SPEED score and NIBUT before and after treatment. We performed a statistical power analysis for both the SPEED score and NIBUT at 3 months after the final treatment session in the control and IPL-MGX groups. The power (1-β) was >0.90 at the level of α = 0.025, and the sample size was sufficient. Statistical analysis was performed with JMP Pro version 15 software (SAS, Cary, NC, USA). All statistical tests were two sided, and a *p* value of <0.05 was considered statistically significant.

## 3. Results

### 3.1. Patient Characteristics

The characteristics of the 43 study subjects with refractory ADDE and mild MGD, including 23 individuals in the IPL-MGX group and 20 in the MGX group, are presented in Table 1. Approximately 40% of patients had Sjögren syndrome or rheumatoid arthritis.

The frequency of other ADDE therapies previously administered is shown in Table 2, with most patients having been treated with diquafosol eyedrops, topical steroids, hyaluronic acid eyedrops, or punctal plugs.

### 3.2. Efficacy of IPL-MGX

The SPEED score was significantly reduced at 4 weeks after the final treatment session compared with baseline in both IPL-MGX group and MGX groups, and this difference was maintained for up to 3 months (Table 3). LLT was significantly increased at both 1 and 3 months after the final treatment session in the IPL-MGX group but not in the control group (Table 3).

Both NIBUT and FBUT were significantly prolonged at both 1 and 3 months after the final treatment session in the IPL-MGX group, whereas they were significantly improved only at 1 month in the control group (Table 3). Changes in interferometric fringe pattern (lipid layer grade) from one typical of aqueous deficiency (Jupiter like) to the normal condition (pearl like) were apparent in 61% and 35% of eyes in the IPL-MGX group as well as in 30% and 5% of those in the control group at 1 and 3 months, respectively, after the final treatment session compared with baseline (Table 4). Lipid layer grade was thus significantly better in the IPL-MGX group compared to the control group at both 1 and 3 months after the final treatment session. The Fluo score had decreased significantly at both 1 and 3 months after the final treatment session in both groups, with the value being significantly lower in the IPL-MGX group than in the control group at both posttreatment assessment points (Table 3).

**Figure 1:**
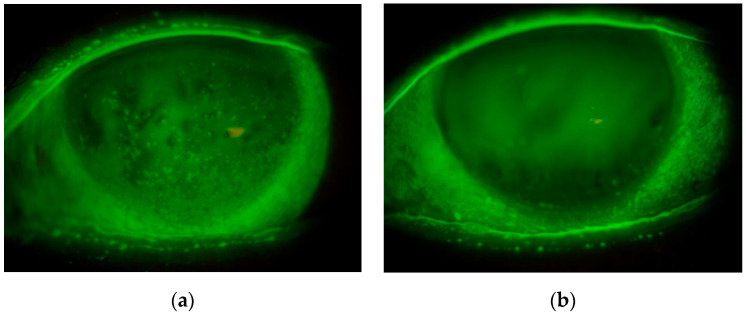
Representative case of a 72-year-old woman treated with intense pulsed light-meibomian gland expression. Slitlamp images of fluorescein staining of the left eye obtained before (**a**) and 3 months after completion of (**b**) treatment showed amelioration of central corneal epitheliopathy. The logMAR visual acuity also improved from 0.15 to 0.09 in association with improvement in the lipid layer of the tear film, whereas tear meniscus height was unchanged.

The logMAR VA of eyes in the IPL-MGX group was significantly improved at both 1 and 3 months after treatment completion compared with baseline, although the values that did not differ between the two groups either before or 1 or 3 months after treatment (Table 5). Eleven of 40 (27.5%) and 12 of 46 (26.1%) eyes in the control and IPL-MGX groups, respectively, manifested central corneal epitheliopathy central corneal epitheliopathy (CCE) with these frequencies not differing significantly between the two groups (*p* = 1, Fisher’s exact test). A significantly improvement in log MAR VA was apparent for the eyes with CCE in the IPL-MGX group compared with those in the control group, both after amerilration of the CCE at 1 month and at 3 months after treatment completion (Table 5, Figure 1).

Meibum grade was significantly decreased in both groups at both 1 and 3 months after treatment completion compared with baseline, whereas vascularity score was significantly decreased at both time points only in the IPL-MGX group (Table 3). Plugging score was significantly decreased in both groups at 1 month after treatment completion compared with baseline, whereas only in the IPL-MGX at 3 months (Table 3). The IPL-MGX group showed a significant difference in meibum grade and lid margin abnormality (plugging and vascularity) scores compared with the control group at both 1 and 3 months after the treatment (Table 3). The meiboscore was not significantly changed at either 1 or 3 months in the IPL-MGX or control group (Table 3). Schirmer’s test value also remained unchanged at 1 and 3 months after the final treatment session in both the IPL-MGX and control groups (Table 3). Finally, TMH was low at baseline and remained so after treatment in both groups (Table 4).

## 4. Discussion

This is the first multicenter study to show an improvement in subjective symptoms and objective signs in patients with refractory ADDE (including those with Sjögren syndrome or rheumatoid arthritis) with mild MGD by treatment with a series of IPL sessions combined with MGX compared to MGX alone. Clinical parameters including ocular symptoms, tear film stability, fluorescein staining, and meibomian gland function were significantly improved by IPL-MGX treatment compared with MGX alone, although tear fluid parameters remained unchanged. Moreover, eyes with CCE showed an improvement in VA associated with IPL-MGX after amelioration of the epitheliopathy. Our results thus suggest that IPL-MGX might be an effective treatment not only for EDE but also for ADDE and mixed EDE-ADDE, although the present study examined only patients with refractory ADDE accompanied by mild MGD. Given that homeostasis of the lipid and tear fluid components of the tear film appears to be important for tear film health, IPL-MGX may have a role as a supportive treatment for ADDE.

We found that IPL-MGX therapy was more effective for the management of refractory ADDE accompanied by mild MGD than was MGX alone, and it was associated with an improvement in meibomian gland-related parameters but not with a change in tear fluid-related parameters. Although >30 studies have indicated that IPL is safe and effective for the treatment of MGD, as far as we are aware only one previous study included patients with Sjögren syndrome [20]. However, this previous study did not evaluate tear fluid-related parameters such as TMH or Schirmer’s test value, but instead assessed only ocular symptoms and meibomian gland expressibility [20]. Five previous studies determined the Schirmer’s test value [30,42,43,44], but all of these studies with the exception of one [43] found no significant change in this parameter in response to IPL therapy. The one exception among these five studies showed that the median Schirmer’s test value increased from 13 to 15 mm (*p* = 0.046) after IPL therapy [43]. The one previous study that measured TMH found no significant difference in this parameter between before and after IPL treatment [21]. Our present results are thus largely consistent with those of previous studies and suggest that IPL does not affect lacrimal glands, but rather influences meibomian glands alone.

With regard to the mechanism of action of IPL in MGD, the treatment likely warms meibomian glands by increasing the temperature of the thin periocular skin and thereby promotes the melting of meibum [16,23]. In addition, the IPL device emits energy that is absorbed by chromophores in hemoglobin and likely thereby promotes closing of abnormal vessels in the lid margin and adjacent conjunctiva as well as attenuates the local release of inflammatory factors from the abnormal vessels [45,46]. A recent study found that the concentrations of various inflammatory factors—including interleukin-17A, interleukin-6, and prostaglandin E_2_—in tear fluid were reduced after IPL therapy [25]. IPL treatment is also likely able to reduce bacterial load of the eyelid margin and the number of *Demodex* mites surrounding adnexa [44] as well as to ameliorate the associated inflammation [47].

Our present results show that MGX alone was also effective for the treatment of refractory ADDE accompanied by mild MGD with regard not only to subjective symptoms but also objective parameters with the exception of LLT, vascularity of lid margins, the meiboscore, TMH and Schirmer value. MGX alone likely does not have anti-inflammatory and meibum-melting effects or improve the condition of the aqueous layer of the tear film. In contrast, IPL-MGX showed significant effects on all of the parameters measured with the exception of the meiboscore, TMH and Schirmer’s test value compared with MGX alone. Our findings suggest that IPL alone might be effective for the treatment of refractory ADDE, although the present study did not examine the effects of IPL without MGX.

The improvement of ADDE by IPL-MGX is consistent with the notion that tear film homeostasis is required for maintenance of tear film health. Self-reported ocular symptoms covered by the SPEED questionnaire were significantly ameliorated after IPL-MGX treatment in the present study, similar to the results of previous studies [20,23,25,29,42,48]. Twenty-two of the 23 study patients (96%) thus showed a decrease in the SPEED score of ≥8 points at both one and three months after the final IPL-MGX treatment session. The Fluo score was also significantly reduced after IPL-MGX therapy, again consistent with previous data [21,23,28,29,30,42,43,44,49]. Of note, eyes with CCE showed a significant improvement in VA after IPL-MGX compared with those receiving MGX alone. The targeting of MGD by IPL-MGX may thus improve the quality and quantity of lipids in the tear film and thereby result in a decline in the concentrations of inflammatory cytokines in tear fluid. Such an action might break the vicious cycle of corneal-conjunctival epitheliopathy and ocular surface inflammation.

The patients treated with IPL-MGX therapy in the present study had experienced ADDE for 8.1 ± 6.7 years (range of 2–24 years), and conventional therapies had proven insufficient of amelioration of ocular symptoms and improvement of tear film–related parameters. A recent study found that most patients with ADDE due to Sjögren syndrome and a disease duration of >3 years also developed MGD, likely because the early destruction of lacrimal glands eventually begins to affect meibomian glands [50]. Indeed, ~40% of patients in the present study also had systemic diseases such as Sjögren syndrome or rheumatoid arthritis. The conditions of such patients may be too severe to manage even with a combination of several conventional therapies. Our study has now demonstrated an improvement in clinical parameters of patients with refractory ADDE and mild MGD by treatment with IPL-MGX or MGX alone, suggesting that therapy targeted to the lipid layer of the tear film may be necessary for such patients who do not respond to conventional therapies. The efficacy of MGX alone in the present study was essentially apparent only one month after treatment completion, whereas that of IPL-MGX remained manifest at three months. The combination of therapies that target both aqueous and lipid layers may thus improve homeostasis of the tear film, resulting in amelioration of corneal-conjunctival epitheliopathy and subjective symptoms as well as an increase in VA.

**Figure 2:**
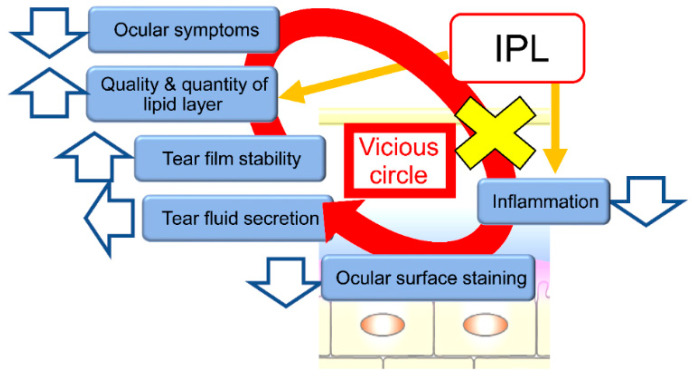
Proposed mechanism of action of IPL therapy on the vicious cycle of dry eye. IPL increases the quality and quantity of the lipid layer of the tear film as well as reduces inflammation of the ocular surface and lid margin. These effects result in amelioration of ocular surface epitheliopathy (ocular surface staining) and an increase in tear film stability, followed by improvement of ocular symptoms without any change in tear fluid secretion. IPL can block the vicious cycle (yellow x) underlying the pathophysiology of dry eye. IPL, Intense pulsed light.

Previous studies have found that ADDE and EDE occur frequently together, given that not only lacrimal glands but also meibomian glands can be affected in ADDE. A reduced production of tear fluid can increase friction between the eyelid and the ocular surface and thereby promote eyelid inflammation [4,5]. We propose that IPL therapy can increase the quality and quantity of lipid in the tear film, dampen the inflammatory reaction due to abnormal vessels, and thereby block the vicious circle underlying the pathophysiology of dry eye. It ameliorates ocular surface epitheliopathy and increases tear film stability, leading to an improvement in ocular symptoms (Figure 2). Together, our results suggest that it is important to treat not only the aqueous layer but also the lipid layer of the tear film in order to restore ocular surface health in patients with ADDE including those with refractory ADDE associated with mild MGD.

Limitations of the present study include its retrospective nature and the relatively small sample size. In addition, both eyes of the study subjects were included, although the two eyes of each patient are not independent. Moreover, the study was not randomized or performed in a masked manner. Finally, osmolarity of tear fluid was not measured as an indicator of the efficacy of IPL-MGX treatment. Our data nevertheless suggest that prospective case-control studies with long-term follow-up are warranted for IPL-MGX treatment of patients with refractory ADDE associated with mild MGD. Further studies should also investigate the effectiveness of such treatment for patients with ADDE alone. Guidelines for IPL therapy based on disease severity are also needed for dry eye patients.

In conclusion, our results suggest that IPL-MGX therapy is effective for patients with refractory ADDE accompanied by mild MGD, with the severe disease of such patients being difficult to manage with conventional therapies. IPL-MGX thus has the potential to improve the condition of not only patients with MGD but also those with refractory ADDE and mild MGD.

## Figures and Tables

**Table 1:** Characteristics of the study subjects with aqueous-deficient dry eye (ADDE) and mild meibomian gland dysfunction in the intense pulsed light (IPL)-meibomian gland expression (MGX) and MGX (control) groups.

Characteristic	Control (MGX) Group (*n* = 20 Subjects, 40 Eyes)	IPL-MGX Group (*n* = 23 Subjects, 46 Eyes)	*p*
Age (years), mean ± SD (range)	61.4 ± 15.1 (31–78)	59.0 ± 15.0 (43–84)	0.64
Sex (male/female)	7/13	9/14	0.78
Duration of ADDE (years), mean ± SD (range)	8.8 ± 5.1 (2–20)	8.1 ± 6.7 (2–24)	0.60
History of contact lens wear	22 eyes of 11 patients	24 eyes of 12 patients	0.85
	(55.0%)	(52.2%)	
Previous ocular surgery	24 eyes of 12 patients	18 eyes of 9 patients	0.17
	(60.0%)	(39.1%)	
Sjögren syndrome	6 patients	7 patients	1
	(30.0%)	(30.4%)	
Rheumatoid arthritis	2 patients	2 patients	1
	(10.0%)	(8.7%)	

*p* values were determined with Mann–Whitney U test (displayed in gray) or Fisher’s exact test. SD, standard deviations.

**Table 2:** Previous therapies for the study patients in the intense pulsed light (IPL)-meibomian gland expression (MGX) and MGX (control) groups.

Therapy	No. (%) of Patients	
	Control Group (*n* = 20)	IPL-MGX Group (*n* = 23)
Diquafosol eyedrops	19 (95.0)	22 (95.7)
Topical steroids	10 (50.0)	21 (91.3)
Rebamipide eyedrops	9 (45.0)	15 (65.2)
Hyaluronic acid eyedrops	8 (40.0)	18 (78.3)
Punctal plugs	8 (40.0)	16 (69.6)
Preservative-free artificial tears	8 (40.0)	10 (43.5)
Omega-3 fatty acid supplementation	3 (15.0)	6 (26.1)

**Table 3:** Characteristics of the study subjects with aqueous-deficient dry eye and mild meibomian gland dysfunction in intense pulsed light (IPL)-meibomian gland expression (MGX) and MGX (control) groups before as well as 1 and 3 months after the final treatment session.

Characteristic	Group	Baseline		1 Month after the Final Treatment Session				3 Months after the Final Treatment Session			
		Mean ± SD	Adjusted *p* Value for IPL-MGX vs. Control	Mean ± SD	Mean Change ± SE	Adjusted *p* Value vs. Baseline	Adjusted *p* Value for IPL-MGX vs. Control	Mean ± SD	Mean Change ± SE	Adjusted *p* Value vs. Baseline	Adjusted *p* Value for IPL-MGX vs. Control
SPEED score	Control	14.2 ± 4.6	0.53	10.1 ± 3.9	−4.2 ± 0.6	<0.001 **	0.003 *	10.4 ± 4.1	−3.8 ± 0.6	<0.001 **	<0.001 **
(0–28)	IPL-MGX	15.9 ± 4.2		6.0 ± 4.6	−9.9 ± 0.7	<0.001 **		4.2 ± 3.9	−11.7 ± 0.9	<0.001 **	
LLT	Control	63.1 ± 16.2	0.72	56.1 ± 11.2	−7.0 ± 2.6	0.055	<0.001 **	57.9 ± 11.8	−5.2 ± 2.7	0.14	<0.001 **
(nm)	IPL-MGX	66.6 ± 24.1		78.6 ± 21.5	14.7 ± 3.2	<0.001 **		84.2 ± 20.6	18.3 ± 2.9	<0.001 **	
Plugging	Control	1.0 ± 0.2	0.88	0.8 ± 0.4	−0.3 ± 0.1	0.002 *	<0.001 **	1.1 ± 0.3	0.1 ± 0.1	0.37	<0.001 **
(0–3)	IPL-MGX	1.0 ± 0.0		0.3 ± 0.4	−0.7 ± 0.1	<0.001 **		0.3 ± 0.5	−0.7 ± 0.1	<0.001 **	
Vascularity	Control	1.7 ± 0.6	0.85	1.7 ± 0.6	0.0 ± 0.0	1	<0.001 **	1.7 ± 0.6	0.0 ± 0.0	1	<0.001 **
(0–3)	IPL-MGX	1.9 ± 0.8		0.8 ± 0.7	−1.1 ± 0.1	<0.001 **		0.9 ± 0.7	−1.0 ± 0.1	<0.001 **	
Meiboscore	Control	1.7 ± 0.6	0.69	1.7 ± 0.6	0.0 ± 0.0	1	0.32	1.7 ± 0.6	0.0 ± 0.0	1	0.69
(0–6)	IPL-MGX	1.5 ± 1.0		1.5 ± 1.0	−0.1 ± 0.0	0.088		1.5 ± 1.0	0.0 ± 0.0	1	
Meibum grade	Control	2.0 ± 0.0	1	1.6 ± 0.5	−0.4 ± 0.1	<0.001 **	<0.001 **	1.9 ± 0.4	−0.2 ± 0.1	0.025 *	<0.001 **
(0–3)	IPL-MGX	2.0 ± 0.0		0.5 ± 0.6	−1.5 ± 0.1	<0.001 **		0.4 ± 0.6	−1.6 ± 0.1	<0.001 **	
NIBUT	Control	2.2 ± 1.1	1	3.6 ± 1.6	1.4 ± 0.2	<0.001 **	0.002 *	2.4 ± 1.2	0.2 ± 0.1	0.22	<0.001 **
(s)	IPL-MGX	2.1 ± 1.3		5.3 ± 2.2	3.2 ± 0.3	<0.001 **		5.5 ± 2.0	3.4 ± 0.3	<0.001 **	
FBUT	Control	2.4 ± 1.1	0.84	3.4 ± 1.5	1.1 ± 0.2	<0.001 **	0.001 *	2.5 ± 1.3	0.1 ± 0.1	0.17	<0.001 **
(s)	IPL-MGX	2.2 ± 1.3		5.2 ± 2.0	3.0 ± 0.4	<0.001 **		5.4 ± 2.1	3.2 ± 0.4	<0.001 **	
Fluo score	Control	3.2 ± 0.8	0.75	2.7 ± 0.9	−0.5 ± 0.1	<0.001 **	<0.001 **	2.9 ± 0.9	−0.3 ± 0.1	<0.001 **	<0.001 **
(0–9)	IPL-MGX	3.4 ± 2.2		0.5 ± 0.9	−3.0 ± 0.3	<0.001 **		0.3 ± 0.6	−3.1 ± 0.3	<0.001 **	
Schirmer’s test value	Control	2.4 ± 1.5	1	2.4 ± 1.5	0.0 ± 0.2	1	1	2.5 ± 1.4	0.1 ± 0.1	1	0.89
(mm)	IPL-MGX	2.3 ± 1.5		2.2 ± 1.4	−0.1 ± 0.2	1		2.2 ± 1.4	−0.1 ± 0.1	0.84	

SPEED score: control group (*n* = 20); IPL-MGX group (*n* = 23). Other characteristics: control group (*n* = 40 eyes); IPL-MGX group (*n* = 46 eyes). *p* values were determined with the Wilcoxon signed-rank test vs. baseline or Mann–Whitney U test vs. control with Bonferroni’s correction (* adjusted *p* < 0.05, ** adjusted *p* < 0.001). SPEED, standard patient evaluation of eye dryness; LLT, lipid layer thickness; NIBUT, noninvasive breakup time; FUBT, fluorescein breakup time; Fluo, fluorescein staining; SE, standard errors; SD, standard deviations.

**Table 4:** Lipid layer grade and tear meniscus height (TMH) of the study subjects with aqueous-deficient dry eye and mild meibomian gland dysfunction in the intense pulsed light (IPL)—meibomian gland expression (MGX) group (*n* = 46 eyes) and MGX (control) group (*n* = 40 eyes) at baseline as well as 1 and 3 months after the treatment completion.

Characteristic		Group	Baseline		1 Month after the Final Treatment Session			3 Months after the Final Treatment Session		
			No. (%) of Eyes	Adjusted *p* Value for IPL-MGX vs. Control	No. (%) of Eyes	Adjusted *p* Value vs. Baseline	Adjusted *p* Value for IPL-MGX vs. Control	No. (%) of Eyes	Adjusted *p* Value vs. Baseline	Adjusted *p* Value for IPL-MGX vs. Control
Interferometric pattern	Jupiter-like	Control	40 (100.0%)	1	28 (70.0%)	<0.001 **	0.016 *	38 (95.0%)	<0.001 **	0.003 *
	Pearl-like		0 (0.0%)		12 (30.0%)			2 (5.0%)		
	Crystal-like		0 (0.0%)		0 (0.0%)			0 (0.0%)		
	Jupiter-like	IPL-MGX	46 (100.0%)		18 (39.1%)	<0.001 **		30 (65.2%)	<0.001 **	
	Pearl-like		0 (0.0%)		28 (60.9%)			16 (34.8%)		
	Crystal-like		0 (0.0%)		0 (0.0%)			0 (0.0%)		
TMH	Low	Control	40 (100%)	1	40 (100%)	1	1	40 (100%)	1	1
	Normal		0 (0.0%)		0 (0.0%)			0 (0.0%)		
	High		0 (0.0%)		0 (0.0%)			0 (0.0%)		
	Low	IPL-MGX	46 (100.0%)		46 (100.0%)	1		46 (100.0%)	1	
	Normal		0 (0.0%)		0 (0.0%)			0 (0.0%)		
	High		0 (0.0%)		0 (0.0%)			0 (0.0%)		

*p* values were determined with the chi-square test vs. baseline and Fisher’s exact test vs. control with Bonferroni’s correction (* adjusted *p* < 0.05, ** adjusted *p* < 0.001).

**Table 5:** Logarithm of the minimum angle of resolution (logMAR) visual acuity of the study subjects with or without central corneal epitheliopathy (CCE) in the intense pulsed light (IPL)—meibomian gland expression (MGX) group (*n* = 12 and 32, respectively) and MGX (control) group (*n* = 11 and 29, respectively) at baseline as well as 1 and 3 months after the final treatment session.

	**Group**	**Baseline**		**1 Month after the Final Treatment Session**				**3 Months after the Final Treatment Session**			
		**Mean ± SD**	**Adjusted *p* Value for IPL-MGX vs. Control**	**Mean ± SD**	**Mean Change ± SE**	**Adjusted *p* Value vs. Baseline**	**Adjusted *p* Value for IPL-MGX vs. Control**	**Mean ± SD**	**Mean Change ± SE**	**Adjusted *p* Value vs. Baseline**	**Adjusted *p* Value for IPL-MGX vs. Control**
Total	Control	0.23 ± 0.22	1	0.23 ± 0.22	0.00 ± 0.00	1	1	0.23 ± 0.22	0.01 ± 0.01	0.8	1
	IPL-MGX	0.22 ± 0.21		0.20 ± 0.21	−0.02 ± 0.01	<0.001 **		0.20 ± 0.21	−0.02 ± 0.01	0.003 *	
Without CCE	Control	0.20 ± 0.24	1	0.19 ± 0.24	−0.01 ± 0.00	0.62	1	0.20 ± 0.23	0.00 ± 0.01	1	1
	IPL-MGX	0.23 ± 0.22		0.22 ± 0.23	−0.01 ± 0.00	0.089		0.22 ± 0.22	−0.01 ± 0.01	0.34	
With CCE	Control	0.31 ± 0.14	0.26	0.31 ± 0.13	0.01 ± 0.01	1	0.036 *	0.32 ± 0.15	0.01 ± 0.02	1	0.030 *
	IPL-MGX	0.19 ± 0.17		0.14 ± 0.16	−0.05 ± 0.01	0.016 *		0.14 ± 0.14	−0.05 ± 0.01	0.016 *	

*p* values were determined with the Wilcoxon signed-rank test vs. baseline or Mann–Whitney U test vs. control with Bonferroni’s correction (* adjusted *p* < 0.05, ** adjusted *p* < 0.001). SE; standard errors, SD; standard deviations.
